# Immune modulator therapy compared with vitrectomy for management of complicated intermediate uveitis: a prospective, randomized clinical study

**DOI:** 10.5935/0004-2749.20200079

**Published:** 2024-02-11

**Authors:** Osama Shalaby, Ahmed Saeed, Mohamed Nagy Elmohamady

**Affiliations:** 1 Ophthalmology department, Faculty of Medicine, Benha University, Benha City, Egypt

**Keywords:** Intermediate uveitis, Vitrectomy, Immunomodulation, Macular edema, Uveíte intermediária, Vitrectomia, Imunomodulação, Edema macular

## Abstract

**Purpose:**

To compare the benefits and side effects of pars plana vitrectomy with those of systemic immune modulator therapy for patients with complicated intermediate uveitis.

**Methods:**

This prospective clinical trial enrolled patients with recurrent intermediate uveitis who exhibited minimal improvement of visual acuity, despite injections of periocular steroids. Twenty patients were randomized to the pars plana vitrectomy group or oral steroid and cyclosporine-A group (10 eyes of 10 patients per group). Follow-up was performed for 24 months to study changes in visual acuity, binocular indirect ophthalmoscopy score, fluorescein angiography, and optical coherence tomography findings.

**Results:**

Visual acuity (logarithm of the minimal angle of resolution) significantly improved from 0.71 to 0.42 (p=0.001) in the surgical group, whereas it improved from 0.68 to 0.43 (p=0.001) in the immune modulator therapy group. Seven patients (70%) in the surgical group gained ≥2 lines, and six patients (60%) in the immune modulator therapy group gained ≥2 lines (p=0.970). Fluorescein angiography and optical coherence tomography studies showed that six of seven pars plana vitrectomy patients who had cystoid macular edema experienced improvement, whereas two patients with diffuse macular edema did not experience improvement. In the immune modulator therapy group, three of six patients with cystoid macular edema did not experience improvement, whereas two patients with diffuse macular edema experienced improvement.

**Conclusions:**

Pars plana vitrectomy and immune modulator therapy resulted in significant improvement in visual function in patients with persistent inflammation secondary to chronic intermediate uveitis. Despite this success, there remains a need for the determination of optimal indications for the use of each modality. Immune modulator therapy was successful for the treatment of diffuse macular edema associated with chronic intermediate uveitis, whereas pars plana vitrectomy was not.

## Abstract (pt)

**Objetivo:**

Comparar os benefícios e efeitos col aterais da vitrectomia via *pars plana* com os da terapia imunomoduladora sistêmica em pacientes com uveíte intermediária complicada.

**Métodos:**

Estudo clínico prospectivo incluiu pacientes com uveíte intermediária recorrente que apresentaram melhora minima da acuidade visual, apesar das injeções perioculares de esteroides. Vinte pacientes foram randomizados para o grupo de vitrectomia via *pars plana* ou esteróide oral e ciclosporina A (10 olhos de 10 pacientes por grupo). O acompanhamento foi de 24 meses para estudar alt erações na acuidade visual, o escore da oftalmoscopia binocular indireta, a angiofluoresceinografia e achados na to mográfica de coerência óptica.

**Resultados:**

A acuidade visual (logaritmo do ângulo mínimo de resolução) melhorou significativamente de 0,71 para 0,42 (p=0,001) no grupo cirúrgico, enquanto melhorou de 0,68 para 0,43 (p=0,001) no grupo da terapia imunomoduladora. Sete pacientes (70%) no grupo cirúrgico ganharam ≥2 linhas e seis pacientes (60%) no grupo da terapia imunomoduladora ganharam ≥2 linhas (p=0,970). Os estudos de angiofluoresceinografia e tomografia de coerência óptica mostraram que seis dos sete pacientes da vitrectomia via *pars plana* que apresentaram edema macular cistóide melhoraram, enquanto dois pacientes com edema macular difuso não apresentaram melhora. No grupo da terapia imunomoduladora, três dos seis pacientes com edema macular cistoide não apresentaram melhora, enquanto dois pacientes com edema macular difuso melhoraram.

**Conclusões:**

A vitrectomia via *pars plana* e a terapia imunomoduladora resultaram em melhora significative da função visual dos pacientes com inflamação persistente secundária a uveíte intermediária crônica. Apesar desse sucesso, continua sendo necessário determinar as melhores indicações para o uso de cada modalidade. A terapia imunomoduladora foi bem sucedida no tratamento do edema macular difuso associado à uveíte intermediária crônica, enquanto a vitrectomia via *pars plana* não foi.

## INTRODUCTION

Intermediate uveitis (IU) is a chronic intraocular inflammatory condition that affects the middle layer of the uveal tract surrounding the vitreous base^(1)^. Histopathologically, it consists of chronic (generally nongranulomatous) vitreous base inflammation, retinal perivasculitis, and microcystoid macular degeneration. The vitreous is the site of greatest inflammation; it shows varying amounts of cells, fibrin, and cellular debris, depending on the severity of inflammation. In some instances (pars planitis), inflammatory exudates (known as “snowbanks”) may form in the inferior vitreous base, over the peripheral retina, and the pars plana^(2,3)^. The inflammatory products might become fibrovascular membranes on the retinal surface that can exhibit bleeding or retinal traction^(3-5)^. In IU, the anterior segment either is clear or shows low-grade inflammation, and the conjunctiva is white and shows no inflammation. In most patients, IU is solely an ocular disorder. However, in a small number of patients, IU is a component of systemic disease, such as sarcoidosis, multiple sclerosis, Whipple’s disease, human T-cell lymphotropic virus infection, or intraocular lymphoma^(6)^. The primary treatment for IU is corticosteroids^(7)^, which can be administered topically, orally, or periocularly^(8)^. In some patients with IU, a favorable therapeutic response has been noted following the use of immune modulator therapy (IMT), such as cyclosporine-A (CsA)^(9)^. Cystoid macular edema (CME) is a common complication of IU that results in significant reduction of visual acuity. Despite rigorous treatment with steroids and other immune modulator therapies, macular edema may persist in a substantial number of patients^(10)^.

Recent advances in vitreoretinal surgery have expanded the indications of vitrectomy for the treatment of several intraocular inflammatory diseases^(11)^. The role of vitreous surgery in IU and uveitic macular edema is uncertain; however, there have been sporadic reports regarding the outcomes of pars plana vitrectomy (PPV) in patients who are unresponsive to medical treatment^(11-13)^. The purpose of this study was to prospectively compare the therapeutic effects of PPV and IMT in patients with chronic IU and secondary macular edema refractory to repeated periocular steroid treatment.

## METHODS

This study protocol was approved by the Benha University research ethics committee. Twenty patients (20 eyes; persistent inflammation mainly in one eye for each patient) with recurrent IU were prospectively recruited between October 2012 and November 2015; follow-up was performed for 24 months. The trial was carried out in the Department of Ophthalmology, Benha University Hospital. The treatment intervention was verbally explained to each patient, and written consent was obtained prior to participation in the study.

Recruitment criteria were persistent or recurrent IU (vide infra) after three periocular steroid injections, with best-corrected visual acuity of 20/40 (0.5) or worse. These patients exhibited complications included macular edema and retinal fibrovascular growths. Recruited patients had no other coexisting ocular pathology or previous intraocular surgery. Patients were assigned to the surgical group (PPV) or IMT group (oral steroid + CsA) (Table 1). Selection of the treatment modality was based on the bilaterality of the disease (IMT group), the presence of epiretinal membranes (ERMs) (surgical group), and the patient’s preference. Both modalities were explained thoroughly and clearly for each patient. In patients with bilateral IU, the more inflamed eye was enrolled in the study.

**Table 1:** Pre-treatment characteristics of patients in the surgical and IMT groups

Characteristics	Surgical Group (10 eyes of 10 patients)	Immune modulator group (10 eyes of 10 patients)
1. Age (years) Range	17-32	16-36
Mean (SD):	26.8 (7)	26.5 (8)
2. Sex:	4 men, 6 women	4 men, 6 women
3. Etiology and Pathology Idiopathic	6 (3 men and 3 women)	6 (2 men and 4 women)
Pars Planitis:	3 (1 men and 2 women)	3 (1 men and 2 women)
Multiple sclerosis	1 (1 woman)	NA
4. Visual acuity (logMAR) Range	0.5-1.0	0.4-1.0
Mean (SD)	0.71 ± 0.3	0.68 ± 0.3
5. AC activities (range)	0-1+	0-2+
6. Vitritis (BIO score) (range)	2-4	2+-4+
7. OCT evaluation: Cystoid macular edema	4 (2 men/2 women)	3 (1 man/2 women)
Diffuse macular edema	2 (2 women)	2 (1 man/1 woman)
Epiretinal membranes	3 (2 men/1 woman)	1 (1 woman)

SD= standard deviation; BIO= binocular indirect ophthalmoscopy; OCT= optical coherence tomography.

Clinical examination included assessment of Snellen visual acuity, measurement of intraocular pressure by Goldmann applanation tonometry, and examination of the globe (anterior segment and fundus) by slit lamp biomicroscopy. Inflammatory activity in the anterior chamber was assessed by the presence of cells and flares and was graded on a 0-4 scale^(1)^. Inflammatory activity in the vitreous was assessed by the presence of cells, debris, and snow banking (in patients with pars planitis). Vitreous inflammation was graded in accordance with the Nussenblatt method using a binocular indirect ophthalmoscopy (BIO) score system, based on the clarity of the fundus details^(1,14)^. The BIO score ranges from 1 to 4; a score of 1 is characterized by a few vitreous cells, minimal haze, and clearly visible posterior pole (i.e., minimal vitritis). In contrast, a score of 4 is characterized by unclear fundus details (i.e., severe vitritis)^(14)^. For macular status evaluation, all patients were subjected to thorough clinical fundus biomicroscopy, fluorescein angiography, and optical coherence tomography (OCT) examination. The grading protocol used for identification of CME from fluorescein angiography was identical to that of the Early Treatment Diabetic Retinopathy Study, in which CME was identified by the accumulation of dye in petaloid or honeycomb-like spaces within the retina in late angiography phases^(15)^. OCT (ZEISS Cirrus™ HD-SD-OCT Model 4000) images were evaluated by a masked reader and reviewed for the presence or absence of macular edema (cystoid/diffuse). CME was determined and graded in accordance with the protocol of Ouyang et al.^(16)^ Cystoid spaces on OCT B-scans were defined as circular or ovoid intraretinal hyporeflective spaces present at the same approximate transverse location on two adjacent B-scans. CME was graded as present, questionable, or absent. When the edema did not exhibit the same pattern as cystoid edema and was present in multiple areas on OCT (but not all frames, potentially due to eye movement), it was defined as diffuse macular edema (DiME). OCT was also used to explore other retinal pathologies, such as ERMs. Patients with best-corrected visual acuity worse than 20/40 (0.5) with annoying floaters (e.g., vitritis of 2+ or worse) and macular edema with or without ERMs were recruited for the study.

In the surgical group, patients underwent PPV via the three-port technique using microsurgical techniques to peel ERMs. Surgeries were performed for two patients under local anesthesia and for eight patients under general anesthesia. Three patients underwent peripheral retinal photocoagulation in the areas of snow banking and retinal holes. All surgical patients had a short course of topical postoperative prednisolone, which was tapered over 2-3 weeks. Patients in the IMT group received oral steroid (prednisolone) at a dose of 1 mg/kg body weight. The dose was subsequently tapered over 3 months to a final dose of 10-20 mg prednisolone/day. Prednisolone was combined with oral CsA (Neoral^®^; Novartis Pharmaceuticals Corporation) at a dose of 3-5 mg/kg/day; CsA dose was adjusted on the basis of the clinical response and to control the trough blood concentration (target level: 100-250 ng/mL). Clinical examinations were repeated at 1 week, 1 month, and 3 months post-treatment; they were then repeated every 3 months until 24 months post-treatment. OCT scanning was performed before treatment; it was then performed at 1 week, 1 month, and 3 months post-treatment, followed by every 3 months until 24 months post-treatment.

The three parameters for determination of treatment success or failure were: visual acuity, vitritis (BIO score), and fluorescein angiography/OCT changes during the specified follow-up visits and at the end of the study period.

### Statistical analysis

Snellen visual acuity values were converted to the logarithm of the minimal angle of resolution (logMAR) for statistical analysis, as well as for scatter plot representation. Relationships between variables were evaluated using paired Student’s t-tests. Two-tailed distributions were used to compare outcomes between both groups, including changes in visual acuity, OCT findings, and vitreous inflammatory activity; in addition, 95% confidence intervals were calculated. All tests of association were considered to be statistically significant when p<0.05.

## RESULTS

At the beginning of the study period, bilateral IU (mild-moderate severity) was observed in six patients in the IMT group; this increased to eight patients (80%) during the course of follow-up. The study focused on one eye in each patient: the eye that was more severely affected at the time of recruitment; however, both eyes benefited from systemic treatment. In the surgical group, mild IU developed in three patients (30%) during the follow-up period, but these occurrences did not warrant treatment. An additional patient in the surgical group developed active IU in the non-operated eye, with deteriorated vision (0.4) that required periocular triamcinolone-A injection to relieve the inflammation.

### Subjective outcomes: visual results

The preoperative mean visual acuity (logMAR) in the surgical group was 0.71 (Table 1). At 24 months after PPV, mean visual acuity (logMAR) in the surgical group had significantly improved to 0.42 (p=0.001); five eyes (50%) exhibited vision of 0.5 or better (Table 2). Seven patients (70%) exhibited significant improvement in visual acuity of ≥2 Snellen lines (Table 2). In six of seven patients with preoperative CME, the visual acuity improved markedly after PPV and ERM peeling. Visual acuity did not improve postoperatively in two patients in the surgical group (both had DiME) (Table 2).

**Table 2:** Demographic baseline and 24 months post management data of the recruited patients

No	Age	Sex	Eye	Snellen VA^*^	Baseline 2Yrs	Bio-score		Macular edema		Management
						PreT	PostT	PreT	PostT	
1	31	F	LE	0.3	0.8	2	0	CME	Improved	Surgical
2	18	F	LE	0.2	0.8	3	0	Q		Surgical
3	28	F	RE	0.1	0.1	4	1	DiME	No change	Surgical
4	32	M	RE	0.1	0.16	3	1	CME	No change	Surgical
5	29	M	LE	0.25	0.6	3	0.5	CME	Improved	Surgical
6	30	M	LE	0.25	0.8	3	1	CME	Improved	Surgical
7	17	F	RE	0.2	0.5	3	0	CME	Improved	Surgical
8	24	F	LE	0.5	0.14	4	1	DiME	No change	Surgical
9	19	F	RE	0.2	0.4	4	1	CME	Improved	Surgical
10	30	M	RE	0,16	0.32	4	1	CME	Improved	Surgical
11	36	M	RE	0.2	0.4	3	2	DiME	Improved	1MT
12	29	F	RE	0.1	0.16	2	2	CME	No change	1MT
13	16	M	RE	0.4	1.0	3	1	CME	Improved	1MT
14	17	F	LE	0.4	0.8	2	0	NME		1MT
15	32	F	RE	0.16	0.1	4	2	CME	No change	1MT
16	31	M	LE	0.32	0.8	3	0.5	CME	Improved	1MT
17	22	F	LE	0.2	0.25	4	3	CME	Improved	1MT
18	23	M	LE	0.16	0.4	3	1	DiME	Improved	1MT
19	25	F	RE	0.2	0.6	4	1	NME		1MT
20	24	F	RE	0.16	0.16	4	4	CME	No change	1MT

CME= cystoid macular edema; DiME= diffuse macular edema; F= female; IMT= immune modulator therapy; LE= left eye; M= male; PreT= pre-treatment; PostT= post-treatment; Q= query macular edema; RE= right eye; SG= surgical group; VA= visual acuity.

* **Snellen visual acuity.**

In the IMT group, baseline visual acuity (logMAR), was 0.68 (Table 1); at the end of the follow-up period, visual acuity (logMAR) had significantly improved to 0.43 (p<0.005). Five (50%) eyes reached a vision of 20/40 or better, and vision improved by ≥2 lines in six patients (60%). Mean visual acuity in the IMT group did not improve in three patients with CME but improved in two patients with DiME. Vision did not improve in one patient who had severe floaters (Table 2).

**Figure 1:**
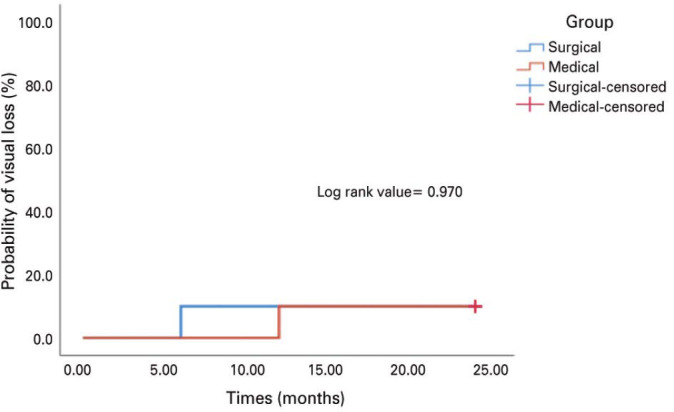
Kaplan-Meier curves of the probability of loss of two lines in visual acuity during the follow-up period.

There was no significant difference between the two groups regarding visual improvement of ≥2 lines. Moreover, there was no significant difference between two Kaplan-Meier curves (for the medical and surgical group) regarding the probability of loss of two lines of visual acuity during the follow-up period (log-rank p=0.970) (Figure 1).

### Objective outcomes: fluorescein angiography/OCT

Quantitative and qualitative assessments of the macula were performed in all patients both preoperatively and postoperatively. Fluorescein angiography and OCT studies showed that nine patients in the surgical group (who did not receive periocular steroids) had macular edema. Seven of these patients had CME and ERMs; the other two patients had DiME (Table 1). ERMs were successfully peeled in three patients in the surgical group. Six patients (60%) with CME showed significant improvement after surgery. Of the remaining four patients, one with persistent CME did not show improvement; another with query macular edema showed marked improvement postoperatively (Table 2). Finally, two patients with DiME (20%) did not show improvement (Table 2) despite surgical treatment.

In the IMT group, four of 10 eyes (40%) showed one or more clinical signs of persistent inflammation. Six patients (60%) responded positively to IMT and showed both clinical and photographic improvement. Three eyes showed mainly persistent CME; one patient with severe vitritis and CME did not show improvement despite the use of IMT, whereas two patients with DiME showed improvement.

The BIO score reflects the degree of vitreal opacities; this improved markedly in the surgical group after mechanical removal of the vitreous. Three patients exhibited a lower postoperative BIO score; however, they did not experience parallel vision enhancement. BIO scores showed varying extents of improvement in the IMT group (Table 2).

### Complications of surgery

The most common postoperative complication following vitrectomy was the development of cataract; two eyes developed progressive cataracts requiring extraction within 6 months postoperatively, whereas one eye developed a mild and stationary cataract that was not surgically removed. Three patients exhibited transient ocular hypertension, which was treated with topical anti-glaucoma agents for 4 weeks. During PPV surgeries, epiretinal gliosis and retinal tear each occurred in one patient.

### Complications of medications

Most patients in the IMT group showed good tolerance to IMT; however, two patients developed gastrointestinal disturbances due to oral CsA, which were managed by dose adjustment and discussion with the patient. Three patients developed complicated (i.e., posterior subcapsular) cataracts during the follow-up period; one was sufficiently dense to require cataract extraction at 3 months after initiation of systemic oral steroid therapy.

## DISCUSSION

IU is typically a mild to moderate inflammatory disea se with a favorable course and prognosis. Indications for IU treatment are visual acuity of 20/40 (0.5) or worse, the presence of substantial floaters, or a type of retinal complication due to persistent inflammation. CME, epiretinal fibrovascular membranes, vitreous hemorrhage, and dense vitreous debris are the most common causes of visual impairment in IU patients. CME is typically a sequela of chronic intraocular inflammation, and its persistence depends on the duration and severity of inflammation.

There are many therapeutic approaches for patients with complicated IU. Treatment is typically provided in a sequential manner. Topical, periocular, intravitreal implant, or systemic steroids are the initial treatments for symptomatic IU or impaired vision. Immunosuppressive agents are added if the initial treatment fails to control inflammation^(17-19)^. PPV has been used for treatment of chronic IU, especially when associated with CME and/ or ERMs^(4,20-22)^.

In our prospective clinical trial, we compared two treatment modalities for patients with persistent IU that did not respond to the initial use of periocular steroid injections. In patients with complicated IU, deteriorated vision improved by ≥2 lines in 70% of the eyes treated with vitrectomy, compared with 60% of the eyes treated with IMT; however, this difference was not statistically significant. Fluorescein angiography and OCT helped to determine the reasons for failed visual acuity improvement in the remaining patients in both groups. In a study of 16 patients (18 eyes) with chronic IU, randomized for either PPV or IMT, Karina et al. found that nine of 11 eyes (82%) treated with PPV showed resolution of inflammation. In contrast, four of seven eyes (57%) treated with IMT exhibited persistent inflammation requiring subsequent PPV. The authors of the abovementioned study did not investigate the impacts of DiME on either treatment modality, nor did they investigate its impacts on visual outcomes. Tranos et al. evaluated the efficacy of PPV in the management of chronic uveitis with CME in 23 eyes of 23 patients^(22)^; 12 patients underwent PPV, whereas 11 patients received systemic corticosteroid and/or IMT during the study period. In the surgical group, mean visual acuity (logMAR) significantly improved, from 1.0 at baseline to 0.55 at 6 months after vitrectomy (p=0.011). Conversely, mean logMAR in the IMT group improved by only 0.03 (p=0.785); this marginal improvement was due to the persistence of stable CME in four eyes and deterioration of CME in two eyes. In our patients, the effects of PPV were encouraging with regard to the clinical outcome and OCT measurements in eyes with recalcitrant IU; in particular, 70% of the eyes showed improvement that remained at 24 months postoperatively. Although removal of the vitreous may influence visual outcomes, the exact mechanisms by which PPV causes improvements in IU are unclear. There is some evidence that removal of inflammatory mediators (cytokines) from the vitreous gel may have resulted in the reduction of antigen presentation and interruption of the vicious inflammatory cycle in the diseased macula^(23-25)^. Furthermore, removal of the vitreous may have improved vision through improvements in media clarity^(26)^. Notably, removal of ERMs will eliminate mechanical traction on the macular surface, thereby enabling the retina to regain its normal anatomical architecture^(26)^. Nevertheless, one patient with DiME did not exhibit visual improvement, and a second patient showed deteriorated vision due to DiME persistence postoperatively. Presumably, there is a minimal therapeutic effect of PPV on the retina in patients with DiME because the mechanisms of edema formation are unique in these patients. In patients with CME, asso ciated ERMs were observed; removal of the tension produced by these membranes may have contributed to the resolution of CME. In DiME, the inflammation is diffuse, confined to internal retinal layers, and does not manifest on the retinal surface; therefore, blood-borne systemic drugs may be more likely to reach an area of tissues that is effectively wider than the region that can be impacted by mechanical therapy. In addition, there were complications from PPV (three patients developed cataract), one patient developed epiretinal gliosis, and one patient developed an intraoperative retinal tear that was sealed using an endolaser. Therefore, although the surgical approach is effective, there remain limitations. IMT is effective in the management of chronic IU that is resistant to treatment with steroid alone, but some patients do not respond to IMT due to permanent adverse changes in the retina. In our study, three patients with CME in the IMT group did not show improvement; this was presumably because of the delay in patient presentation, which increased structural damage and may have led to permanent macular degeneration. Those patients presented to the uveitis clinic at ≥3 months after the last periocular injection. Another patient did not show improvement in visual acuity because of marked media opacities. The pathological mechanisms underlying the ineffectiveness of IMT in these patients may also involve the presence of ERMs with continuous cytokine production. In addition, fibrovascular and gliotic tissues exert a mechanical (pulling) effect on the surface of the retina. Notably, the two patients with DiME responded to IMT; this observation should receive closer attention, because it suggests that IMT can reach and spread into the retinal microenvironment.

In the IMT group, we combined CsA with oral steroids at the initiation of treatment because the previous therapeutic regimen (periocular steroids alone) failed to control the inflammation. There are known complications related to the extended administration of steroids; therefore, we greatly restricted the use of the drug (a maximum dose of 10-20 mg/day) and then primarily pursued treatment with CsA. The additive effect of CsA, the T-lymphocyte inhibitory drug (anti-calcineurin), may be the underlying source of improved vision in 60% of patients in the IMT group^(27)^. CsA complications were minimal, probably because of the relatively young age of patients who received this treatment.

There were some limitations in this study. First, the sample size was small, because of the relatively low number of patients who develop this type of uveitis. A previous history of periocular steroid injection could have introduced bias, as the outcomes in these patients may not be entirely due to the effects of surgery or IMT. However, all patients had chronic IU that was refractory to previous local treatment; therefore, the course of steroid injections presumably did not have a substantial effect on the functional and anatomic results in this study. A larger randomized study, with a longer follow-up period, is needed to determine the relative contributions of PPV and IMT to the management of IU, based on the subtypes or complications in patients with this condition. Notably, there is an ongoing multi-center uveitis steroid treatment trial to compare systemic anti-inflammatory therapy versus fluocinolone acetonide implant treatment for the management of IU. The preliminary results of this multi-center uveitis steroid treatment trial indicated that neither approach was superior and that selection between these treatments should be performed on the basis of each patient’s particular circumstances^(28,29)^.

In our study, we compared the efficacies of two therapeutic modalities, PPV versus IMT, for the treatment of recalcitrant IU; we assessed their impacts on visual acuity, BIO score, and OCT parameters for a follow-up period of 24 months. The results we have presented support the findings of previous work, in that PPV has beneficial effects for visual function in patients with CME secondary to chronic IU. Visual recovery was accompanied by improvement of CME in OCT assessments; this visual recovery was significant relative to preoperative levels. The timing of surgery is crucial for its success, as in other instances of retinal pathology management. Non-surgical options for the treatment of chronic IU are useful, particularly those involving IMT; in our study, this was evidenced by the failure of surgery to resolve IU in patients with DME. Therefore, surgical treatment in these patients may be not preferable, especially in the absence of retinal surface tension due to vitreous membranes. The decision to perform surgical management should be made if no improvements are observed in clinical and OCT signs, despite maximum doses in IMT. Although this study lacked robust data, similar to other pilot studies, our findings are sufficient to justify the performance of a large-scale trial with a long follow-up period, which could be used to define the appropriate indications for PPV and IMT in patients with complicated IU. This future study should incorporate advancements in modern OCT and other diagnostic tools to precisely determine when each treatment modality should be applied.
